# Electromyographical Comparison of Four Common Shoulder Exercises in Unstable and Stable Shoulders

**DOI:** 10.1155/2012/783824

**Published:** 2012-08-07

**Authors:** Aaron Sciascia, Nina Kuschinsky, Arthur J. Nitz, Scott D. Mair, Tim L. Uhl

**Affiliations:** ^1^The Shoulder Center of Kentucky, Lexington Clinic, Lexington, KY 40504, USA; ^2^Sport Gesundheitspark Berlin, 13347 Berlin, Germany; ^3^Department of Rehabilitation Sciences, University of Kentucky, Lexington, KY 40536, USA; ^4^Department of Orthopaedic Surgery and Sports Medicine, University of Kentucky, Lexington, KY 40536, USA

## Abstract

This study examines if electromyographic (EMG) amplitude differences exist between patients with shoulder instability and healthy controls performing scaption, prone horizontal abduction, prone external rotation, and push-up plus shoulder rehabilitation exercises. Thirty nine subjects were categorized by a single orthopedic surgeon as having multidirectional instability (*n* = 10), anterior instability (*n* = 9), generalized laxity (*n* = 10), or a healthy shoulder (*n* = 10). Indwelling and surface electrodes were utilized to measure EMG activity (reported as a % of maximum voluntary isometric contraction (MVIC)) in various shoulder muscles during 4 common shoulder exercises. The exercises studied effectively activated the primary musculature targeted in each exercise equally among all groups. The serratus anterior generated high activity (50–80% MVIC) during a push-up plus, while the infraspinatus and teres major generated moderate-to-high activity (30–80% MVIC) during both the prone horizontal and prone external rotation exercises. Scaption exercise generated moderate activity (20–50% MVIC) in both rotator cuff and scapular musculature. Clinicians should feel confident in prescribing these shoulder-strengthening exercises in patients with shoulder instability as the activation levels are comparable to previous findings regarding EMG amplitudes and should improve the dynamic stabilization capability of both rotator cuff and scapular muscles using exercises designed to address glenohumeral joint instability.

## 1. Introduction

 Glenohumeral instability is a common shoulder condition and has been defined as the inability to maintain the humeral head in the glenoid fossa [1]. Instability can present due to a traumatic or atraumatic mechanism in one or multiple directions [1, 2]. The prevalence of primary dislocations is equally distributed between patients above and below 45 years of age however the incidences of recurrent dislocations are primarily in a younger population [3]. Initial intervention for shoulder instability is temporary activity modification and the implementation of a shoulder strengthening program [4, 5]. Strengthening the rotator cuff is thought to be critical due to its role of stabilizing the humeral head within the glenoid fossa [6, 7]. The effectiveness of strengthening shoulder muscles has been demonstrated using a series of exercises performed primarily with the arm below 45° of shoulder elevation [4]. However, specific exercises have been identified that facilitate high EMG activity of the rotator cuff and scapular musculature that require humeral motions at or above 45° of shoulder elevation [8–14].

 The identified exercises not only elicit high levels of muscular activity in healthy individuals but also simulate functional activities. The prone external rotation exercise at 90° of abduction targets activation of the infraspinatus [8, 13] and mimics the act of throwing a ball [15]. Elevation of the arm in the plane of scapula is a functional movement utilized in everyday life (i.e., reaching for an object on a shelf) and has been found to target the deltoid, supraspinatus, and serratus anterior musculature [8, 14]. Prone horizontal abduction has been found to target the supraspinatus and deltoid musculature [8, 13, 14] and would simulate placing or reaching for an object away from the body. The serratus anterior muscle has been identified as an important scapular stabilizer and is often targeted via the push-up plus maneuver [12, 16]. Although these exercises simulate functional activities and appear to produce high EMG activity in healthy populations, it is unclear if the same exercises will generate similar activation levels in persons with glenohumeral instability.

Glenohumeral muscular activity in individuals with shoulder instability has been found to be altered in both amplitude levels and duration of activation compared to the muscle activity of healthy individuals during active elevation. Deltoid muscle activity (duration/amplitudes) has been found to be 10–38% lower, while rotator cuff muscle activity has been 15–20% higher in persons with shoulder instability [17–19]. Elevated muscle activity/duration is thought to be a compensation by the dynamic stabilizer for compromised static restraint [18], whereas lower muscular activity may arise from pain-derived inhibition or the subconscious limitation of movement to avoid positions where subluxation and/or dislocation occur [20].

Previous studies have typically examined muscle activity between patients with only one type of shoulder instability and healthy subjects [17–19]. However, the aforementioned shoulder rehabilitation exercises are often prescribed to strengthen shoulder musculature for all types of shoulder instability [5, 21]. The effectiveness of these exercises in targeting specific shoulder and scapular musculature is well established in healthy subjects but limited information exists regarding the effect of these rehabilitation exercises in individuals with various forms of shoulder instability. Therefore, the purpose of this study was to determine if these four traditional shoulder rehabilitation exercises would activate the shoulder muscles in subjects with various types of shoulder instability similarly to subjects with otherwise stable shoulders.

## 2. Materials and Methods

### 2.1. Subjects

 Thirty nine subjects (11 males and 28 females, age = 21 ± 3.3 years, height = 172 ± 9.6 cm, and weight = 73 ± 19.4 kg) volunteered for this study. All volunteers were evaluated and classified by a single orthopedic surgeon into one of four categories (healthy = H, generalized laxity = GL, multidirectional instability = MDI, and anterior instability = AI). Subjects were excluded from this study if they had any neurological disorders present or had previous shoulder surgery. All participants signed consent forms approved through the Institutional Review Board at the University of Kentucky.

 Healthy subjects (*n* = 10) were individuals with no history of shoulder injury, no shoulder instability, no generalized laxity, and no pain with activities of daily living (ADLs). Subjects with evidence of having a loose glenohumeral joint were classified into the generalized laxity group (*n* = 10) if they possessed at least 3 of 5 generalized laxity findings described by Carter and Wilkinson [22], as well as no pain with ADLs, no history of shoulder “giving way,” and no previous shoulder subluxation or dislocation. Subjects were classified with multidirectional instability (*n* = 10) based on (1) verbal history or recurrent subluxation and/or dislocation; (2) history of the sensation of the shoulder “giving way”; (3) positive findings for all of the following tests: load and shift test, apprehension test, relocation test, and sulcus sign. Subjects were classified as having anterior instability (*n* = 9) based on (1) pain with ADLs; (2) history of painful recurrent subluxation and/or dislocation; (3) history of the sensation of the shoulder “giving way”; (4) positivity for the anterior apprehension test and relocation test. Both multidirectional and anterior instability were diagnosed in a binary (yes or no) fashion.

### 2.2. Instrumentation

For the upper trapezius, middle deltoid, and serratus anterior, two bipolar Ag/AgCl surface electrodes (Medicotest, Olstykke, Denmark) were placed on the skin with an interelectrode distance of 2 cm and parallel to the muscle fibers. The skin was prepared by shaving and lightly debriding the skin with fine grade sandpaper, and vigorously cleaning with an alcohol swab prior to electrode placement to minimize skin impedance [23]. The electrodes were place according to standardized locations [23]. For the supraspinatus, infraspinatus, and teres, major two sterile bipolar fine-wire electrodes were inserted about 1 cm apart in the muscle belly with a 27 gauge hypodermic needle using a double needle insertion technique [24].

All electrodes were connected to the data acquisition unit; a Myopac unit (Run Technologies, Mission Viejo, CA). The Myopac unit has a common mode rejection ratio (CMRR) of 90 dB and transmitted the raw EMG data via a fiber optic cable to its receiver unit at 1000 Hz. The analog signal was converted to digital via a PCI 1200/12 bit A/D board. All surface EMG raw data were band pass filtered at 20–500 Hz and indwelling EMG channels were band pass filtered at 10–1000 Hz. The EMG data was acquired, analyzed, and stored using the Datapac 2000 Version 2.33 software (Run Technologies, Mission Viejo, CA). In order to determine specific arcs of motion, a specially designed photocell sensor system was used and synchronized to EMG data for all data collection. A photocell was adhered to a portable wall with hook and loop material at 30° intervals. The arc of motion was confirmed prior to data collection with an electronic inclinometer (Dualer, JTech Medical, Salt Lake City, UT, USA). A small handheld flashlight (MiniMaglite, Ontario, Canada) was held by each subject as the arm was moved through the arc of motion during each open chain exercise, while for the push-up plus, it was attached to each subject's thoracic spine with an elastic strap or clipped to a sports bra. The push-up plus exercise was divided only into an eccentric phase, lowering the torso and a concentric phase, elevating torso away from table.

Exercise resistance was standardized to a load of 25% of maximal exerted force. For the identification of the maximal exerted force, a hand-held dynamometer (Jtech Medical, Salt Lake City, UT) was utilized as previously reported [25]. Three maximal contraction trials applied in the midrange of each exercise were averaged to determine the 25% load used during resistance exercises [25]. The mean load used during the scaption exercise was 2 ± 0.7 kg, while the mean load for the prone horizontal abduction exercise (PHA) was 1.5 ± 0.5 kg and the mean load for prone external rotation (PER) was 1.9 ± 0.8. For the push-up plus (PUP) exercise, no additional weight was added.

### 2.3. Testing Protocol

Subjects performed manual muscle testing in previously described standardized positions known to activate each of the 6 muscles studied [26, 27] in order to normalize all EMG data to maximal voluntary isometric contraction (MVIC) for comparisons between groups [28]. Subjects performed two maximal contractions for 5 seconds with 60 seconds of rest between each trial [26]. All subjects were able to maintain the testing positions for each MVIC with no subject reporting giving way of the shoulder during collection.

**Figure 1:**
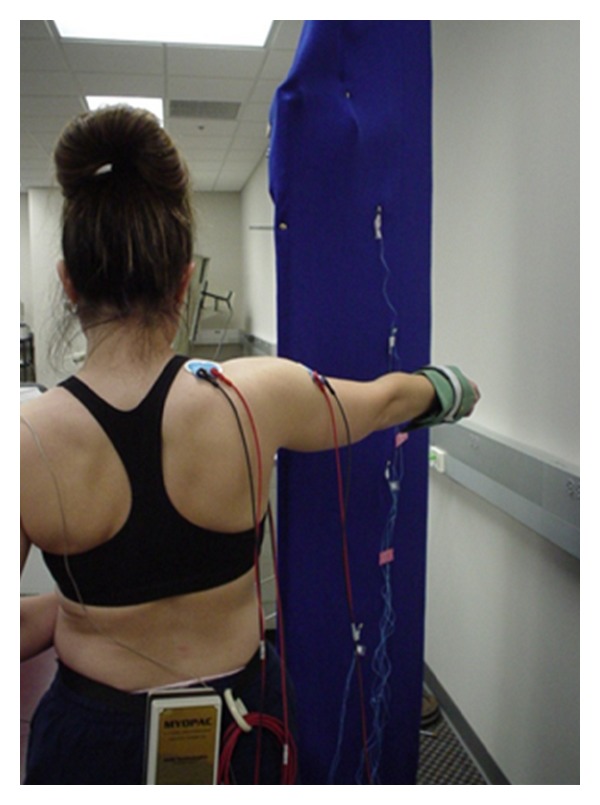
Illustration of the performance of scaption exercise with subject aligning their arm motion with the portable wall and the photo cells aligned in 30° arcs.

**Figure 2:**
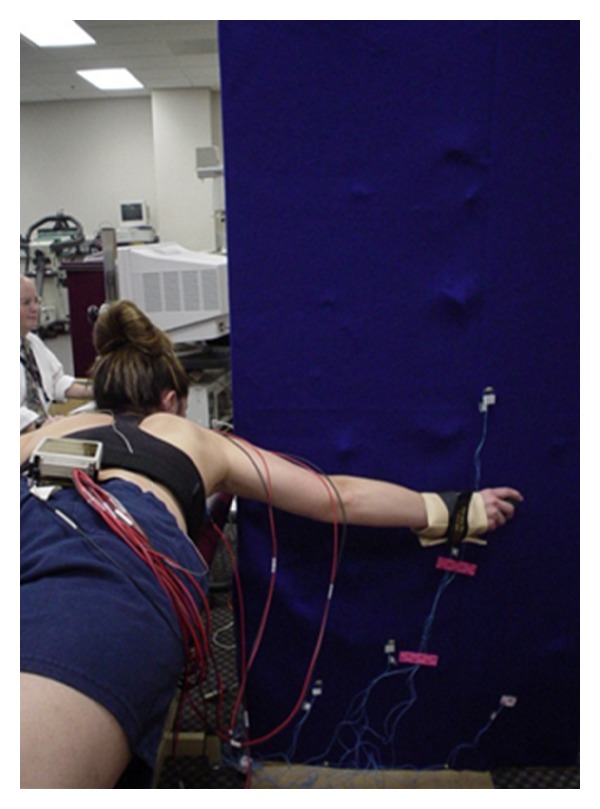
Illustration of the performance of the prone horizontal abduction exercise with shoulder externally rotated.

Following MVIC acquisition, each subject performed six repetitions of the four rehabilitation exercises. These particular exercises were chosen due to their common usage in rehabilitation programs reported in the literature [8, 12–14]. Scaption with external rotation was performed standing with the elbow fully extended starting the exercise at the side [12, 14]. The subject elevated the arm to 120° and then lowered it to the side. A portable wall was used to keep the arm in the scapular plane (45° anterior to the frontal plane) (Figure 1). PHA was performed with the subject lying prone, arm hanging perpendicular to the floor off the side of the table, subject's arm was elevated to 90° with thumb pointed up, and then lowering back to the starting perpendicular position [8, 13, 14] (Figure 2). PER at 90° of abduction was performed with subject lying prone and with forearm hanging perpendicular to the floor off the side of the table [8, 13]. The upper arm was supported on the table with a towel to keep the humerus aligned with the midline of the subject's body. The PUP was performed with the subject on his/her hands and knees. Starting with the elbows fully extended and scapula protracted, the subjects lowered themselves to 90° of elbow flexion, representing the eccentric phase of the exercise and then returned to the starting position, considered the concentric phase [12]. The order of exercises was randomized by pulling the exercise names out of an envelope by each subject prior to starting data collection. A three minute rest was given between each exercise in order to minimize fatigue and order bias [29]. The three open chain exercises were controlled for velocity (50°/s) of motion with a metronome since EMG amplitudes are affected by velocity [30]. The push-up plus was performed at subjects' self-selected pace which approximated 1 repetition per 2 seconds. Prior to data collection all subjects familiarized themselves to the exercise and rate of motion to assure they moved smoothly and that the flashlight was activating the photocells to demark the arcs of motion.

### 2.4. Data Reduction

 All EMG data collected during the exercises performed were represented as (%MVIC) and the three middle trials of the six repetitions were averaged together to represent muscle performance. The arcs of motion were demarked by the photocell voltage deflection that was triggered as the light passed over it. Maximal EMG activity or 100% MVIC is represented by the highest measured root mean square (RMS) amplitude during a 500 milliseconds (ms) time window during the two MVICs. The RMS amplitude for each arc of motion was determined for each muscle. This RMS amplitude recorded for each arc was divided by the maximal RMS amplitude for the particular muscle [28]. The average of the three repetitions represented a subject's muscular performance for the exercise. This average was combined with all other subjects in their group to be used for later statistical analysis.

### 2.5. Statistical Analysis

The EMG data was analyzed for normal distribution but was found not to be normally distributed. Therefore, to determine if EMG amplitude differences between each group existed, multiple Kruskal-Wallis tests were used which is a nonparametric test comparable to the parametric analysis of variance test. For this cross-sectional study, EMG amplitudes (%MVIC) were separately compared between groups (H, GL, MDI, and AI) for each of the four exercises, muscles tested, and arc of motion. Analyses were performed for each muscle separately to account for the different “pick up area” of the indwelling and surface electrodes. If significance was found, a post hoc analysis using a pair wise Mann-Whitney *U* test was applied to determine where differences existed between groups. Significance was set, a priori, at *P* ≤ 0.05 for both analyses. All statistical analyses were performed using SPSS version 18 (IBM, Armonk, NY). To facilitate interpretation of EMG amplitude results, previous literature has categorized EMG amplitudes as low (<20% MVIC), moderate (20–50% MVIC), and high (>50% MVIC) which will be used in this study [31, 32].

## 3. Results

Scaption exercise descriptive analysis is presented in Table 1. During the concentric arc of motion, 60–120° generated the moderate EMG activity levels (20–50% MVIC) for nearly all muscles studied except for the teres major. There was a significant difference in EMG activity between the groups for one phase during the eccentric 60–30° arc in the teres major that generated very low amplitudes. Post hoc analysis revealed that the GL group generated more EMG activity than the healthy and MDI groups (*P* ≤ 0.02). The very low amplitudes ranging from 0 to 4% MVIC suggest these statistically significant differences are unlikely to be clinically relevant. During the 60–120° concentric arcs of motion, the H group appeared to generate more EMG activity in the serratus anterior compared to the other groups but was not found to be significant (*P* = 0.09) as was the case for all other muscles studied between the four groups for this exercise.

PHA descriptive analysis is presented in Table 2. The supraspinatus and infraspinatus were primarily activated at the moderate to high levels (30–70% MVIC) during this exercise in the 30–90° arcs. Post Hoc analysis revealed that the GL group was significantly more active in the infraspinatus compared to the H and AI groups between 30 and 90° arc concentrically (*P* < 0.02) and during the 90–60° arc eccentrically (*P* < 0.005, Table 2). Additionally, GL was significantly more active than the MDI group during the 30–60° arc concentrically (*P* = 0.05). It should be noted that only 4 out of the 9 AI subjects and 8 out of the 10 MDI subjects reached the 60–90° arc of motion as they felt a sense of instability or inadequate strength to obtain this amount of motion. There was also significant less EMG amplitudes in the teres major during the 30–60° concentric phase in the MDI group compared to the AI and H groups (*P* < 0.02, Table 2).

The PER exercise descriptive analysis is presented in Table 3. The primary muscle activated during this exercise was the infraspinatus. During the concentric 60–90° arc of motion, the highest levels of activity ranging from 60 to 90% MVIC occurred for all groups studied. The infraspinatus activity in the MDI and GL group trended towards greater activity during this arc of motion but was not significant (*P* = 0.07). Overall there was no significant differences between groups for any of the muscles studied with one exception for the infraspinatus in the eccentric 60–30° arc (*P* = 0.04). Post hoc analysis revealed that the GL and AI groups generated significantly more EMG activity than the healthy group (*P* < 0.02, Table 1).

 The PUP descriptive analysis is presented in Table 4. There were no significant differences between the four groups for either the concentric or eccentric phase of the push-up plus. The primary muscle activated with this exercises is the serratus anterior which generates high levels (>50%MVIC) of EMG activity relative to the other muscles studied.

## 4. Discussion

The primary purpose of this cross-sectional study was to determine if the four rehabilitation exercises would activate the shoulder muscles in subjects with shoulder instability similarly to subjects without instability. We defined type of instability using a single orthopedic surgeon clinical examination into one of four categories: multidirectional instability, anterior instability, generalized joint laxity, or healthy normal shoulders. Although there were limited differences between groups, the overall majority of this study's findings supports previous research established in a healthy population, that these shoulder rehabilitation exercises target specific musculature [8, 12–14]. This study adds to the literature by demonstrating that the target musculature is activated to basically the same level in an unstable population. Additionally, this study identified some potentially clinically relevant differences that should be considered when prescribing these rehabilitation exercises for patients with unstable shoulders. The specific exercises will be discussed below.

### 4.1. Scaption

Scaption elicited moderate to high muscle activity levels from all muscles and all groups tested which is in agreement with prior research [8, 14]. This was particularly true in the arcs of motion from 60° to 120° suggesting that scaption would be an ideal exercise to implement in order to strengthen multiple muscles simultaneously especially when preparing a patient to perform challenging tasks requiring open chain elevation beyond shoulder level.

Kronberg et al. [18] reported a nonsignificant increase in rotator cuff activity in subjects with anterior instability and subjects with generalized laxity during abduction in the frontal plane. Similarly, we found a similar pattern of increased rotator cuff activity in subjects with shoulder laxity which was not significantly different between groups. It is possible that in persons with unstable shoulders, the ligaments and capsule which are typically lax, can affect the mechanism of concavity-compression [33, 34] placing greater emphasis on the dynamic stabilization capability of the rotator cuff muscles. This would imply that clinicians should attempt to increase or improve dynamic stabilization by addressing muscle performance of the rotator cuff muscles in individuals with a lax shoulder.

 The GL, MDI, and AI groups had variable serratus anterior, but equal levels of upper trapezius activation during the 60–120° arcs of scaption when compared to the normal group. The serratus anterior muscle assists in upward rotation of the scapula during arm elevation [27]. The upper ranges of elevation are known to be difficult to achieve in persons with shoulder instability as these ranges of motion tend to provoke episodes of instability. Paletta et al. [35] described an arthrokinematic variation in subjects with shoulder instability where the humeral head would migrate superiorly during active arm elevation limiting the full range of motion from being achieved. The arthrokinematic variation may exist from tissue derangement [35] or from muscles acting out of phase or with increased activity (substitution) in an attempt to avoid provocative positions of motion [19, 36, 37]. The relatively slightly lower serratus anterior activity in subjects with either instability or generalized laxity might be indicative of the muscular inhibitions described by Paletta et al. [35] which were suggested to occur as a means of limiting the uncomfortable positions of motion. Although the scaption exercise evoked the second highest amount of serratus anterior muscle activity, clinicians should approach the implementation of the maneuver as a global exercise rather than a specific measure for solely strengthening the serratus anterior due to the potential for muscular inhibition in the presence of instability.

### 4.2. Prone Horizontal Abduction

 Prior research has determined prone horizontal abduction with external rotation to be an optimal exercise for activating the supraspinatus in healthy shoulders [8, 13, 14]. After averaging muscle activity of the supraspinatus across all arcs and comparing all exercises, we found that PHA effectively activated the supraspinatus, infraspinatus, and middle deltoid muscles (38–74% MVIC). This finding is in agreement with previous research in healthy population who found that PHA is an ideal exercise to activate these same muscles [8, 13, 14].

There was significantly greater infraspinatus activity in the GL groups compared to the AI and H groups between the exercise arcs of 30–60°, 60–90°, and 90–60°. Due to likely presence of increased glenohumeral joint laxity in multiple directions, the increase in activity in the GL is likely representative of a coping strategy attributed to a need for increased stability in patients with hyperlaxity to center the humeral head in the glenoid [34, 38, 39]. This trend was observed in MDI subjects also but due to the high variability was not found to be significant. Interestingly, 56% of the subjects in the AI group (5 of 9) could not obtain the terminal range of motion for this exercise due to discomfort and inadequate strength. Yet as a group, AI subjects achieved similar muscle activity levels as the healthy group. This observation suggests that this group may have been able to generate activity levels similar to the MDI and GL groups if it were not for pain inhibition. Inability to achieve terminal range for AI subjects indicates that a modification of the PHA may be appropriate. Perhaps, an exercise that does not require as much external rotation would be more appropriate for this type of patients. For the purpose of the study, we attempted to control the position of humeral external rotation to allow for comparisons but in a clinical situation, the exercise would have been modified in order to allow the patient to perform without discomfort and through a greater range of motion. Although scapular kinematics were not measured during this study, previous research has found that a protracted scapula reduces force generating capabilities in the shoulder [40, 41]. Therefore, it is recommended that clinicians give consideration to scapular position at the start of PHA and scapular kinematics during the PHA exercise to potentially increase pain-free motion during this exercise in AI patients.

### 4.3. Prone External Rotation

 Previous studies have found that prone external rotation at 90° of abduction is an exercise that primarily activates the infraspinatus in healthy shoulders [8, 13]. Ballantyne et al. [42] found that subjects with shoulder pathology had significantly greater infraspinatus activity during PER. Statistically, the current study indicated that there was no significant difference between groups (*P* = 0.07). However, there was a strong trend that the GL and MDI group generated more infraspinatus activity in the 30–90° arcs of the concentric phase of the exercise which is consistent with results from Ballantyne et al. [42] and their EMG amplitudes of 80–85%MVIC are nearly identical to the current study's amplitudes. In the current study, the eccentric phase of the exercise was examined and we found that the GL and AI groups activated their infraspinatus to a higher level than the healthy group (*P* < 0.02). Although there are methodological and population differences between the current study and Ballantyne et al. [42] study, the overall behavior supports their conclusion that alterations in muscular activation levels are likely to appear in pathological shoulders, which included various diagnoses not limited to glenohumeral instability alone. The increased activation in the infraspinatus is likely due to increased demands on this muscle to stabilize the humeral head on the glenoid as suggested by Cain et al. [38] from their cadaveric modeling of posterior shoulder musculature when the ligamentous stabilizers are compromised. Based on the current study's findings, prone external rotation appears to be a good exercise to activate the infraspinatus in both healthy and unstable shoulders. Clinicians should be alert to fatigue during performance of this exercise due to the high muscular demand observed in this study and may need more sets with fewer repetitions to allow for muscular recovery when prescribing this exercise.

### 4.4. Push-Up

 The PUP exercise elicited the greatest amount of muscle activity from the serratus anterior compared to all other muscles. This is concurrent with the findings of Hardwick et al. [10], Decker et al. [16], and Moseley et al. [12] who found that the PUP exercise is a demanding exercise for the serratus anterior. The rotator cuff and scapular muscles were activated equally between all groups and between both phases of the exercise.

### 4.5. Limitations

 There were notable limitations in our study. The 3 types of shoulder laxity (generalized laxity, anterior instability, and multidirectional instability) were classified clinically but were not verified with imaging. However, a single board certified orthopedic surgeon who specialized in treating shoulder pathology performed all screenings prior to a subject being classified into a study group. Our subject population size was low. We tested 39 subjects with approximately 10 subjects in each of four groups. Prior studies which were similar to our design have used 15–20 subjects per group [25, 36, 42]. Kronberg et al. [18] reported differences between pathologic and nonpathologic subjects by testing only 6 subjects per group. Therefore we concluded that 10 per group would be adequate to find differences, though due to the large EMG amplitude variances this was not confirmed in the current study. The large variances observed in this study may be in part due to small number of subjects in each group. There is also a moderate likelihood that fatigue contributed to the large variances recorded in EMG amplitudes. Even though we allotted three minutes between each exercise, all subjects reported that the push-up plus exercise was very demanding and created more fatigue than was expected at the outset of the study. Future studies should increase the number of subjects and require a greater rest period between exercises.

## 5. Conclusion

 Clinicians rehabilitating unstable shoulders are using exercises such as the ones studied here to facilitate dynamic stabilization in the presence of compromised static stabilizers. This study demonstrates that exercises, previously documented to optimally activate glenohumeral and scapulothoracic musculature in stable shoulders, are sufficient in targeting the same muscles during rehabilitation of patients with unstable shoulders. The greatest EMG amplitude for all muscles always occurred during the concentric terminal 30° arc of the exercises. Clinicians should use caution when loading the terminal ranges while performing the PER and PHA exercises as some subjects may not be able to achieve this position with moderate resistive loads. Overall there were few differences in EMG amplitudes of the shoulder muscles between the four groups studied which is most likely due to the large variance in amplitudes. These findings should help clinicians in prescribing appropriate exercises for patients with unstable shoulders.

## Figures and Tables

**Table 1:** Scaption descriptive analysis with mean (Mn) and standard deviation (SD) values reported divided into eight 30° arcs. The results of each Kruskal-Wallis test are presented at the end of each column for the respective muscle and arc.

	0–30°		30–60°		60–90°		90–120°		120–90°		90–60°		60–30°		30–0°	
	Mn	Sd	Mn	Sd	Mn	Sd	Mn	Sd	Mn	Sd	Mn	Sd	Mn	Sd	Mn	Sd
Supraspinatus																
H	23	(10)	33	(12)	40	(12)	41	(12)	31	(9)	25	(7)	19	(6)	11	(5)
GL	27	(10)	39	(12)	46	(15)	46	(14)	35	(12)	29	(7)	22	(4)	14	(4)
MDI	24	(11)	32	(10)	40	(12)	42	(13)	31	(9)	25	(8)	18	(8)	11	(7)
AI	22	(12)	33	(18)	36	(17)	38	(18)	28	(14)	23	(12)	18	(10)	11	(9)
*P* =	0.73		0.37		0.39		0.71		0.53		0.24		0.56		0.36	
Infraspinatus																
H	11	(4)	20	(7)	27	(11)	29	(13)	20	(8)	16	(6)	12	(5)	6	(3)
GL	18	(10)	29	(14)	42	(19)	47	(20)	34	(21)	28	(16)	19	(10)	10	(6)
MDI	10	(12)	21	(18)	33	(29)	39	(33)	25	(22)	19	(20)	12	(15)	5	(11)
AI	14	(4)	24	(8)	32	(11)	37	(13)	28	(11)	23	(11)	15	(7)	8	(3)
*P* =	0.17		0.37		0.28		0.42		0.30		0.26		0.22		0.22	
Teres major																
H	1	(4)	2	(4)	2	(6)	2	(6)	1	(4)	0	(4)	0	(3)	0	(3)
GL	10	(25)	13	(25)	6	(6)	8	(11)	4	(5)	4	(4)	4	(3)	3	(3)
MDI	1	(2)	2	(3)	2	(2)	4	(6)	5	(9)	2	(5)	0	(2)	0	(2)
AI	1	(1)	2	(1)	2	(2)	3	(2)	2	(1)	1	(1)	1	(1)	1	(1)
*P* =	0.32		0.11		0.38		0.51		0.54		0.24		0.02^∗^		0.06	
Middle deltoid																
H	11	(7)	20	(12)	33	(15)	47	(14)	31	(9)	19	(6)	12	(6)	6	(4)
GL	9	(5)	16	(7)	25	(14)	36	(19)	24	(12)	16	(8)	9	(5)	4	(3)
MDI	8	(5)	19	(10)	32	(12)	39	(12)	24	(6)	17	(6)	12	(7)	4	(3)
AI	9	(4)	18	8	30	(12)	43	(19)	27	(11)	18	(6)	11	(3)	5	(3)
*P* =	0.90		0.94		0.53		0.44		0.33		0.87		0.59		0.32	
Upper trapezius																
H	23	(12)	33	(14)	40	(16)	43	(12)	30	(8)	22	(8)	16	(7)	8	(8)
GL	21	(10)	34	(17)	41	(21)	41	(15)	29	(11)	23	(11)	16	(6)	10	(5)
MDI	18	(7)	27	(13)	37	(18)	39	(20)	27	(14)	20	(11)	14	(7)	8	(6)
AI	14	(8)	25	(10)	35	(13)	38	(16)	25	(13)	18	(8)	12	(5)	6	(4)
*P* =	0.16		0.55		0.87		0.79		0.43		0.81		0.36		0.32	
Serratus anterior																
H	24	(14)	42	(21)	59	(20)	63	(18)	42	(17)	29	(13)	14	(12)	5	(5)
GL	20	(18)	33	(22)	46	(23)	49	(21)	32	(15)	24	(13)	13	(12)	5	(7)
MDI	12	(7)	24	(10)	38	(11)	44	(15)	28	(9)	20	(6)	10	(7)	4	(5)
AI	13	(13)	27	(22)	37	(22)	45	(27)	28	(15)	19	(12)	10	(10)	3	(4)
*P* =	0.23		0.21		0.09		0.09		0.23		0.33		0.84		0.66	

^∗^Indicates significant difference between groups.

H: Healthy.

**Table 2:** Prone horizontal abduction descriptive analysis with mean (Mn) and standard deviation (SD) values were reported to be divided into six 30° arcs. The results of each Kruskal-Wallis test are presented at the end of each column for the respective muscle and arc.

	0–30°		30–60°		60–90°		90–60°		60–30°		30–0°	
	Mn	Sd	Mn	Sd	Mn	Sd	Mn	Sd	Mn	Sd	Mn	Sd
Supraspinatus												
Healthy	15	(9)	32	(17)	48	(18)	32	(13)	16	(10)	7	(6)
GL	31	(19)	59	(31)	74	(30)	49	(20)	31	(15)	14	(9)
MDI	20	(14)	39	(22)	52	(27)	33	(16)	20	(11)	7	(9)
AI	16	(10)	32	(15)	45	(16)	27	(10)	18	(11)	6	(5)
*P* =	0.13		0.11		0.08		0.06		0.11		0.12	
Infraspinatus												
Healthy	19	(7)	32	(12)	36	(10)	26	(7)	20	(9)	11	(5)
GL	29	(12)	48	(14)	68	(21)	50	(18)	32	(10)	17	(7)
MDI	20	(15)	38	(24)	57	(42)	39	(25)	23	(13)	12	(13)
AI	18	(9)	30	(18)	33	(16)	24	(7)	21	(11)	11	(6)
*P* =	0.09		0.04^∗^		0.006^∗^		0.003^∗^		0.06		0.16	
Teres major												
Healthy	8	(8)	15	(12)	18	(15)	13	(10)	9	(8)	7	(6)
GL	6	(9)	16	(24)	27	(44)	27	(50)	14	(22)	10	(20)
MDI	2	(2)	2	(3)	5	(4)	5	(4)	3	(3)	2	(3)
AI	3	(3)	6	(4)	10	(8)	8	(8)	6	(3)	3	(2)
*P* =	0.28		0.04^∗^		0.23		0.43		0.43		0.23	
Middle deltoid												
Healthy	11	(6)	26	(10)	46	(20)	33	(15)	15	(7)	8	(4)
GL	11	(5)	26	(14)	43	(28)	28	(17)	16	(7)	10	(5)
MDI	9	(6)	25	(14)	38	(6)	25	(6)	17	10	10	(7)
AI	12	(5)	32	(16)	53	(25)	32	(12)	21	(9)	11	(4)
*P* =	0.55		0.6		0.66		0.6		0.56		0.45	
Upper trapezius												
Healthy	4	(4)	11	(8)	23	(18)	16	(10)	7	(5)	3	(2)
GL	7	(3)	14	(6)	19	(9)	14	(11)	10	(7)	6	(6)
MDI	6	(9)	11	(19)	11	(11)	6	(7)	8	(15)	5	8
AI	4	(3)	10	(6)	22	(13)	14	(10)	8	(6)	5	(3)
*P* =	0.16		0.16		0.13		0.16		0.28		0.36	
Serratus anterior												
Healthy	4	(7)	6	(9)	7	(13)	4	(5)	3	(5)	3	(4)
GL	4	(5)	6	(6)	11	(8)	8	(5)	6	(5)	2	(3)
MDI	2	(3)	4	(5)	6	(10)	4	(6)	2	(3)	2	(3)
AI	2	(3)	3	(4)	5	(6)	3	(3)	3	(3)	2	(3)
*P* =	0.73		0.4		0.09		0.06		0.14		0.89	

^∗^Indicates significant difference between groups.

**Table 3:** Prone external rotation descriptive analysis with mean (Mn) and standard deviation (SD) values were reported to be divided into six 30° arcs. The results of each Kruskal-Wallis test are presented at the end of each column for the respective muscle and arc.

	0–30°		30–60°		60–90°		90–60°		60–30°		30–0°	
	Mn	Sd	Mn	Sd	Mn	Sd	Mn	Sd	Mn	Sd	Mn	Sd
Supraspinatus												
Healthy	9	(8)	14	(13)	21	(16)	16	(12)	8	(9)	5	(6)
GL	10	(10)	19	(17)	29	(19)	18	(12)	11	(11)	14	(9)
MDI	8	(6)	13	(11)	20	(17)	16	(14)	9	(9)	4	(7)
AI	8	(13)	14	(17)	30	(27)	21	(22)	10	(13)	7	(15)
*P* =	0.68		0.56		0.47		0.88		0.92		0.83	
Infraspinatus												
Healthy	16	(6)	31	(9)	63	(22)	33	(12)	13	(5)	5	(2)
GL	26	(11)	50	(19)	83	(19)	49	(16)	24	(12)	11	(9)
MDI	18	(17)	42	(31)	83	(63)	49	(38)	20	(18)	8	(15)
AI	18	(8)	37	(15)	56	(10)	35	(9)	20	(7)	8	(5)
*P* =	0.14		0.08		0.07		0.13		0.04^∗^		0.4	
Teres major												
Healthy	3	(5)	3	(5)	7	(7)	4	(5)	2	(4)	5	(6)
GL	5	(7)	6	(7)	8	(9)	7	(8)	5	(7)	5	(5)
MDI	1	(2)	2	(1)	3	(2)	1	(2)	1	(1)	2	(2)
AI	4	(5)	7	(9)	8	(10)	6	(7)	6	(8)	6	(8)
*P* =	0.3		0.61		0.45		0.33		0.17		0.5	
Middle deltoid												
Healthy	5	(4)	4	(4)	6	(6)	4	(4)	3	(3)	3	(3)
GL	5	(5)	6	(7)	8	(10)	5	(5)	3	(2)	3	(2)
MDI	3	(3)	4	(4)	4	(3)	3	(2)	2	(2)	2	(2)
AI	6	(5)	6	(6)	12	(9)	9	(6)	5	(4)	4	(4)
*P* =	0.57		0.86		0.4		0.22		0.77		0.39	
Upper trapezius												
Healthy	2	(3)	3	(4)	4	(4)	2	(3)	1	(3)	0	(2)
GL	3	(3)	5	(5)	7	(5)	7	(10)	3	(3)	2	(2)
MDI	3	(5)	5	(5)	7	(5)	4	(4)	5	(11)	2	(6)
AI	3	(4)	5	(5)	8	(7)	6	(7)	3	(6)	2	(4)
*P* =	0.85		0.59		0.14		0.09		0.36		0.57	
Serratus anterior												
Healthy	5	(5)	9	(5)	20	(14)	13	(9)	5	(4)	3	(5)
GL	4	(4)	10	(9)	27	(22)	15	(10)	5	(4)	1	(1)
MDI	3	(4)	7	(6)	14	(10)	9	(7)	3	(3)	1	(2)
AI	3	(3)	8	(8)	23	(25)	14	(15)	5	(6)	1	(2)
*P* =	0.83		0.58		0.51		0.6		0.75		0.73	

^∗^Indicates significant difference between groups.

**Table 4:** Push-up Plus descriptive analysis with mean (Mn) and standard deviation (SD) values reported in two arcs. The results of each Kruskal-Wallis test are presented at the end of each column for the respective muscle and arc.

	Concentric		Eccentric	
	Mn	Sd	Mn	Sd
Supraspinatus				
Healthy	23	(15)	22	(14)
GL	26	(14)	28	(16)
MDI	21	(27)	17	(18)
AI	24	(13)	22	(10)
*P* =	0.53		0.31	
Infraspinatus				
Healthy	29	(13)	20	(11)
GL	29	(14)	36	(24)
MDI	31	(15)	23	(11)
AI	30	(14)	22	(10)
*P* =	0.97		0.40	
Teres major				
Healthy	10	(12)	7	(10)
GL	9	(8)	10	(11)
MDI	28	(37)	30	(31)
AI	11	(15)	5	(6)
*P* =	0.59		0.23	
Middle deltoid				
Healthy	23	(12)	14	(7)
GL	16	(12)	15	(12)
MDI	24	(16)	21	(12)
AI	15	(8)	15	(9)
*P* =	0.26		0.41	
Upper trapezius				
Healthy	17	(17)	13	(11)
GL	18	(10)	20	(9)
MDI	15	(16)	15	(16)
AI	16	(11)	15	(11)
*P* =	0.84		0.27	
Serratus anterior				
Healthy	77	(46)	45	(23)
GL	65	(34)	66	(39)
MDI	48	(18)	41	(25)
AI	55	(32)	39	(22)
*P* =	0.43		0.16	
